# Isolated ovarian tuberculosis in an Immuno- competent woman in the post partum period: case report

**DOI:** 10.1186/s13048-018-0472-2

**Published:** 2018-11-22

**Authors:** Wondimu Gudu

**Affiliations:** Department of Obstetrics & Gynecology, Saint Paul’s Hospital Millennium Medical College, P.O.Box 1271, Addis Ababa, Ethiopia

**Keywords:** Ovary, Tuberculosis, Abscess, Pelvic, Isolated

## Abstract

**Background:**

Pelvic tuberculosis is a rare form of extrapulmonary tuberculosis. It commonly involves the fallopian tubes and the uterus from a lympho-hematogeneous spread. The presentation of pelvic tuberculosis as an isolated ovarian abscess is extremely rare and is reported only twice.

**Case presentation:**

a 25 yrs. old para III mother in the post partum period undergone laparotomy for suspected tuboovarian abscess/ovarian tumor after presenting with abdominal pain, pelvic mass and fever. Intra-operatively, Isolated right ovarian mass with caseation in the cavity but no significant pelvic adhesions was detected and right oophorectomy was done. Post operative Histopathology of surgical specimens revealed tuberculous leision and patient recovered well after anti-tuberculosis treatment.

**Conclusion:**

Isolated ovarian tuberculosis is a very rare form of Genital Tuberculosis which should always be considered in the evaluation of a woman presenting with any adnexal mass in highly prevalent areas.

## Introduction

Tuberculosis (TB) is one of the top 10 causes of death worldwide. There were 1.4 million TB deaths and estimated 10.4 million new TB cases in 2015 [1]. Although pulmonary TB is the commonest clinical form, the incidence of extra-pulmonary Tuberculosis (EPTB), particularly abdomino-pelvic TB, is progressively increasing especially in developing countries due to the emergence of HIV/AIDS) [2]. It is estimated that Genital Tb affects about 12% of women with pulmonary Tuberculosis (PTB) and 15 to 20% of women with EPTB [3].

Genital TB is almost always secondary to TB elsewhere in the body, the commonest primary site being the lungs [4]. Lympho-hematogenous dissemination is most common, followed by intra-luminal and neighborhood spread [4, 5].

The commonest sites of involvement are the fallopian tubes in 90–100% and the endometrium in 50–80% of the cases while the ovaries are involved in 20–30% of cases genital Tb [5]. The classical presentation of Female genital tuberculosis is with a triad of infertility, menstrual irregularity & chronic pelvic pain. But isolated ovarian tuberculosis presenting as an adnexal mass and mimicking ovarian tumor is an extremely rare clinical variety and is reported only twice in the literature [6]. In here, a very rare case of an isolated unilateral ovarian tuberculosis in a woman in the postpartum period is presented.

### Case presentation

A 25 years old, married, para III, Ethiopian Somali woman presented with an insidious onset of lower abdominal pain, offensive vaginal discharge and intermittent fever of 1 month duration. She had associated anorexia, vomiting & episodic diarrhoea which were followed by progressive weight loss and drenching night sweats. Two weeks prior to her presentation she developed progressively increasing abdominal distension with urgency, frequency, dysuria and straining at micturition. The woman had smooth vaginal delivery at a health centre 2 months back and claimed to have uneventful pregnancy. She was lactating & didn’t see any menses after delivery. She had no cough and didn’t report any known medical illnesses. She lived with 2 relatives in the same room who were being treated for pulmonary tuberculosis 2 years ago. All her previous pregnancies and deliveries were uneventful. She was repeatedly treated with unspecified antibiotics at local health facilities with no improvement.

On Physical examination the woman was acutely sick looking. The pulse rate was 112 per minute; temperature 38 ^o^c and she had pale conjunctive. The remarkable findings were on abdominal and pelvic examinations. Abdominal examination revealed distended abdomen with lower abdominal tenderness and rebound tenderness. There was a 14 cm by 12 cm sized, firm, tender, pelvic mass with limited mobility. Shifting dullness & fluid thrill were negative and bowel sounds were normal. Speculum examination showed hyperaemic otherwise normal looking cervix. Digital vaginal examination findings were: smooth & firm cervix; bulging pouch of Douglas; firm 18 cm by 18 cm sized pelvic right adnexal mass with adnexal and cervical motion tenderness.

**Fig. 1:**
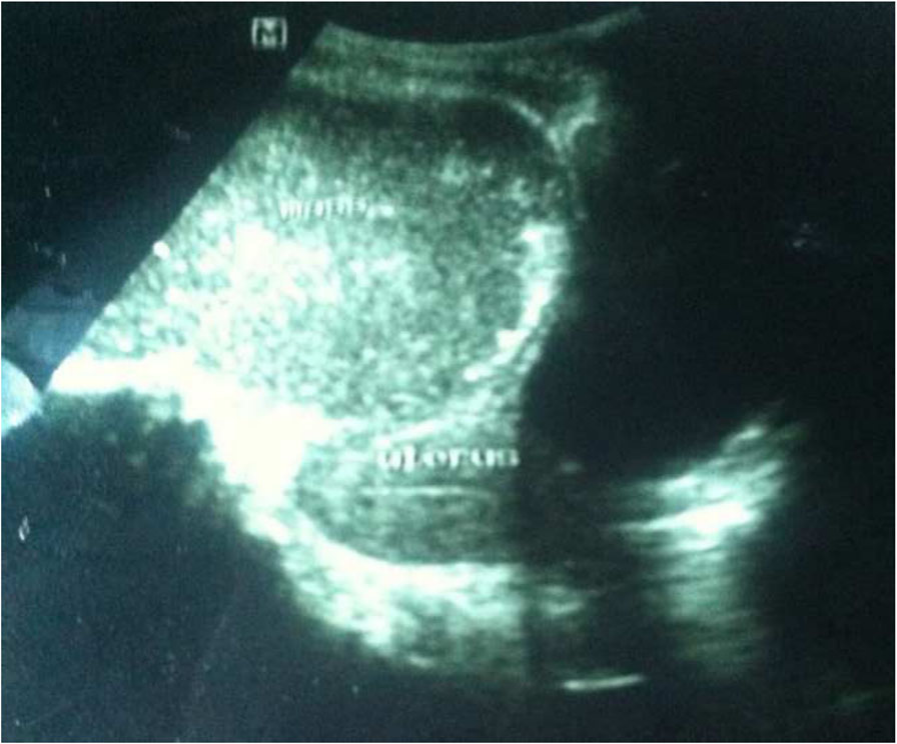
Ultrasound image of the hypo-echoic right adnexal mass

Laboratory tests revealed anemia (Hgb = 9 g/dl), leukocytosis with left shift, raised ESR and Pyuria. Organ function tests, chest x-ray & plain abdominal films were normal. Ultrasound showed a hypoechoic, well outlined, thick-walled pelvic mass in the right adnexa extending to the pouch of Douglas with internal echoes (Fig. 1). Doppler revealed normal flow & resistance pattern of ovarian vessels. The uterus was normal sized with thin endometrial slit. The conclusion was a right adnexal (ovarian) tumor with possible differential diagnosis of tubo-ovarian abscess.

**Fig. 2:**
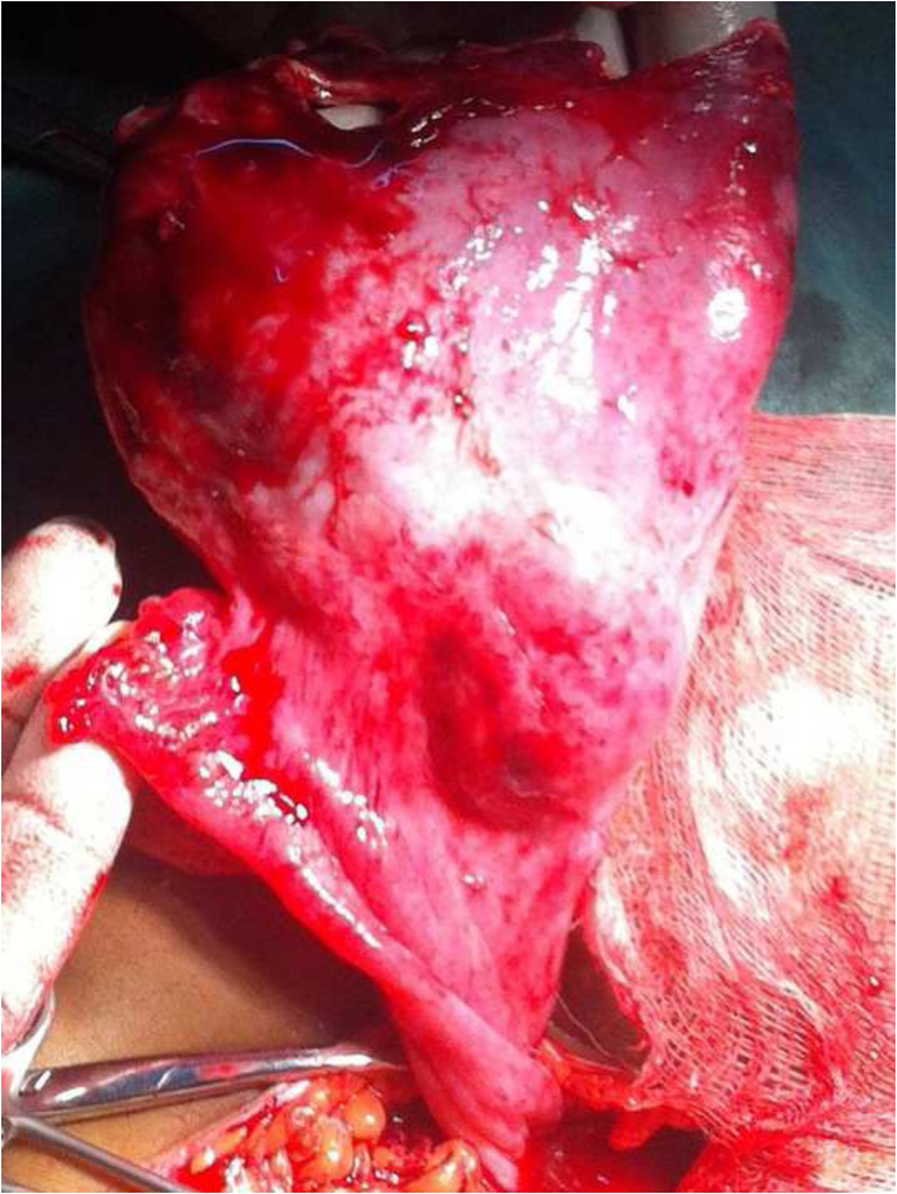
The right ovarian mass after incision made at upper boarder and normal appearing right fallopian tube

**Fig. 3:**
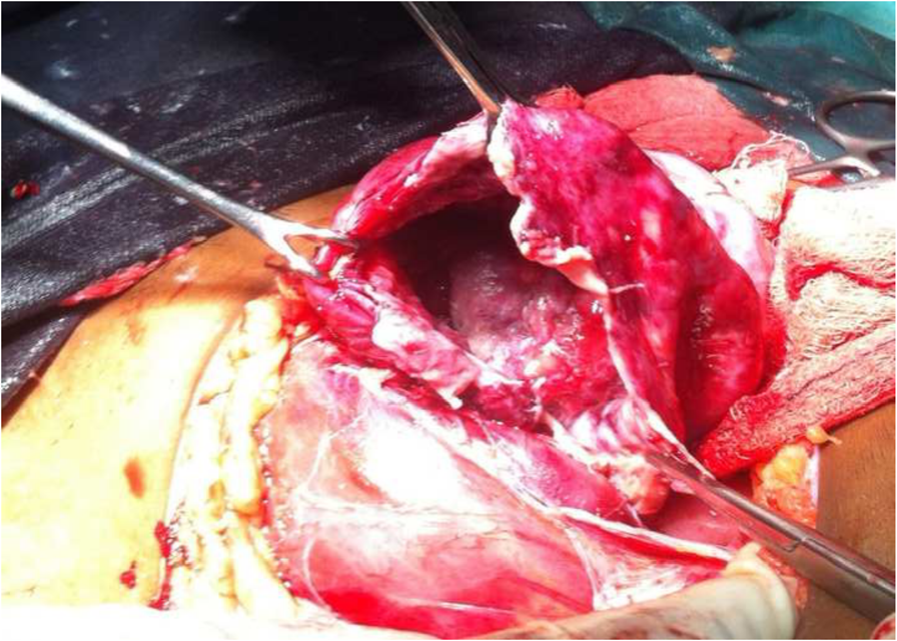
The ovarian sac of abscess and the empty cavity after drainage

The mother was counselled on the presumptive diagnosis and informed consent was obtained for emergency laparotomy which revealed matted omentum obscuring the pelvic cavity & filmy adhesions (involving the uterus, right adnexa, intestines and bladder). Careful dissection and adhesiolysis, revealed thin offensive fluid extruding from the right adnexa; right ovarian 14 cm by 14 cm sized cystic to firm mass located between the ovarian fossa & pouch of Douglas (Figs. 2 and 3). There was no grossly identifiable ovarian tissue. Both tubes appeared normal except for presumably oedematous serosal surface of the right fallopian tube. The uterus and appendix were normal in size & appearance. Procedure was completed after performing right oophorectomy (complete excision of the mass) and securing omental & peritoneal biopsies. Incision of the wall of the mass revealed thick (caseating) pus with no mass in the cavity of the sac. Postoperatively she was having recurrent high grade fever for more than 1 week despite potent antibiotics administration.

The histopathology examination revealed chronic inflammation with caseation and histocytes granuloma formation which was consistent with tuberculous lesion. But culture and immune-histologic analysis were not done because of facility constraints The woman was then put on anti-Tb regimen (category-I) containing INH, Rifampicin, Pyrazinamide and Ethambutol and showed dramatic improvement at her follow up visit after completing the intensive phase.

## Discussion

Female genital tuberculosis (FGTB) is an important cause of significant morbidity especially infertility and chronic pelvic pain. The actual incidence of Genital Tb cannot be determined accurately because at least 11% of patients are asymptomatic and may remain undiagnosed [4, 5].

Several risk factors exist for EPTB and TB of the female genital tract. Most are host factors causing impaired immunity [7, 8]. Increased exposure to the infection is another factor and about 20% of patients with genital TB give a history of TB in their immediate family [4]. This was evident in our case who had prolonged exposure with diagnosed cases of tuberculosis.

Genital tract is vulnerable to tuberculous disease after puberty, and most cases occur during the childbearing period [4]. The focus in the lung often heals, and the lesion may lie dormant in the genital tract for years, only to reactivate at a later time [2, 4]. Although postpartum period is generally a risk factor for ascending infections (PID), we couldn’t find any literature evidence explaining why there was reactivation of genital Tb in the immediate post-partum period in our case that had primarily ovarian involvement with grossly unaffected uterus and tubes. But it is reported that in those 15% of patients who didn’t have infertility, symptoms of genital TB develop in one third to one half within one year after their last pregnancy was completed [4] which was evident in our case.

The clinical presentation of genital TB depends upon the site of involvement of genital tract [4, 5]. The age of presentation in 80% of women is 20–40 years, especially in developing countries [5]. Systemic symptoms tend to be relatively mild, if present, and may include weight loss, fatigue, and mild nocturnal fever. The four major presenting complaints in symptomatic patients are: infertility, abnormal uterine bleeding, pelvic pain, and amenorrhea [4].

Physical examination can be normal in up to 50% of cases of female genital Tb [5]. There is little correlation between presenting complaints and physical findings in genital TB [4]. When abnormal findings are present, they usually consist of adnexal masses or signs of ascites [4, 9].

In the case of Ovarian Tuberculosis, Two clinico-pathological patterns of involvement are described: perio-ophoritis and ooporitis [4]. Peri-ooophorits is the most common form of tuberculous involvement of the ovary in which the tuberculous process starts from the tube and involves the ovary with extension of the lesion from the periphery (tunica) toward the center (parenchyma). This results in a tubo-ovarian mass, which is frequently adherent to omentum and intestines [4]. Oophoritis is a relatively rare condition in which infection starts in the stroma of the ovary, presumably from a hematogenous source that produces a caseating granuloma within the parenchyma. In Tb Oophoritis, typical tubercles or larger foci with caseous centers may be recognized on cross section in the hilum of the ovary [4].

In tubo-ovarian TB, patients usually present with adnexal mass and/or ascites [4, 7, 9]. Tuberculous tubo-ovarian masses are less tender than those due to pyogenic infection, although secondary infection and acute exacerbation may produce sharp pain and tenderness [5, 8]. Fixation of pelvic organs on bimanual examination may be appreciated. In our case, the abdominal and adnexal tenderness can be explained by superinfection of the ovarian mass which was witnessed during laparotomy as there was offensive exudate extruding from the right adnexa.

The clinical findings in most previous case reports of ovarian tuberculosis included: extensive pelvic adhesions; peritoneal seedlings; ascites or multi-septated masses. But the fact that our case presented with an apparent homogenous cystic solitary mass with no adhesions, ascites or peritoneal seedlings and that the presentation was in the postpartum makes it peculiar.

The absence of specific symptoms and conclusive signs during physical examination may delay a proper diagnosis [5, 7]. In ovarian Tb, the clinical presentation cam mimic ovarian malignancy which was also evident in our case in whom a diagnosis of ovarian tumor was made based on clinical and US findings. It should be noted that diagnostic imaging tests are non specific and both US and CT/MRI scan appearances are similar in both ovarian tuberculous abscess and other neoplastic ovarian masses [6]. An elevated CA-125 level is also found in some of the patients with ovarian Tb which further increases the diagnostic dilemma [5, 6, 9].

Laparoscopy and laparotomy are important in the diagnosis of ovarian/tubo-ovarian Tb. Intraoperative frozen sections (if available) are valuable to rule out the possibility of pelvic Tb in patients undergoing surgery for tubo-ovarian masses, especially in highly prevalent areas [6]. Hence keeping in mind the possibility of tubo-ovarian TB is crucial as it will avoid radical/extensive surgery and unnecessary removal of the ovaries for suspected ovarian malignancy in reproductive age women who desire to retain/preserve fertility. In areas with no frozen section, we believe retaining otherwise normal appearing ovaries during laparotomy for suspected ovarian malignancy is advisable.

The definitive diagnosis of pelvic Tb lies in identifying the tubercle bacilli by microbiologic examinations including culture or PCR which can still be negative despite the presence of tuberculous infection [7–9].

The management of pelvic tuberculosis is in general medical (6 to 9 months of anti-tuberculous therapy). Surgery is limited only for those cases that are unresponsive to treatment. Drainage of pyosalpinx and removal of large tubo-ovarian abscesses can be performed followed by anti Tb for better results [10].

In our case the removal of the diseased ovary was justified as it had gross caseation in the parenchyma and as this will decrease the risk of reactivation post operatively. Even though confirmation of the definitive diagnosis by culture could not be made, Postoperative anti Tb was initiated considering the exposure history of the patient, the histopathologic granulomatous lesion, and the high Tb endemicity of the area.

## Conclusions

Isolated ovarian tuberculosis is a very rare form of female genital tuberculosis. It usually presents with adnexal mass and can mimic ovarian tumor. In highly prevalent areas, ovarian tuberculosis should be considered in the evaluation of women with adnexal masses.

### Consent

Written informed consent was obtained from the patient for publication of this case report and any accompanying images.

## Abbreviations

Anti-Tb: Anti-tuberculosis
HIV: Human Immuno-deficiency Virus
PCR: Polymerase Chain Reactions
Tb: Tuberculosis
